# Modeling nearshore-offshore exchange in Lake Superior

**DOI:** 10.1371/journal.pone.0193183

**Published:** 2018-02-15

**Authors:** Paul McKinney, Kathy S. Tokos, Katsumi Matsumoto

**Affiliations:** Department of Earth Sciences, University of Minnesota, Minneapolis, Minnesota, United States of America; University of Vigo, SPAIN

## Abstract

Lake Superior′s ecosystem includes distinct nearshore and offshore food webs linked by hydrodynamic processes that transport water and tracers along and across shore. The scales over which these processes occur and their sensitivity to increasing summer surface temperatures are not well understood. This study investigated horizontal mixing between nearshore and offshore areas of Lake Superior over the 10-year period from 2003 to 2012 using a realistically forced three-dimensional numerical model and virtual tracers. An age tracer was used to characterize the time scales of horizontal mixing between nearshore areas of the lake where water depth is less than 100 m and deeper areas. The age of water in nearshore areas increased and decreased in an annual cycle corresponding to the lake′s dimictic cycle of vertical mixing and stratification. Interannual variability of mixing in the isothermal period was significantly correlated to average springtime wind speed, whereas variability during the stratified season was correlated to the average summer surface temperature. Dispersal of a passive tracer released from nine locations around the model lake’s perimeter was more extensive in late summer when stratification was established lakewide than in early summer. The distribution of eddies resolved in the model reflected differences between the early and late summer dispersal patterns. In the eastern part of the lake dispersal was primarily alongshore, reflecting counterclockwise coastal circulation. In the western part of the lake, cross-shore mixing was enhanced by cross-basin currents.

## Introduction

The nearshore areas of large lakes are valuable water resources that provide water to surrounding communities and habitat for a variety of species. They also are vulnerable to degradation by runoff from the surrounding watershed. Water quality in these critical areas is controlled by horizontal mixing processes that disperse pollutants, nutrients and sediment along shore and across shore, thus predicting the effects of runoff on nearshore areas requires an understanding of the scales over which these processes occur. In this study, we evaluate the variability of horizontal mixing (mixing, hereafter) in Lake Superior using a realistically forced three dimensional model of lake hydrodynamics and virtual tracers.

Lake Superior is the world’s largest lake by surface area and the headwaters of the Laurentian Great Lakes. With an average depth of ~150 m, it is the deepest of the Great Lakes and contains approximately ten percent of the world’s surface freshwater, roughly the same amount as the other four Great Lakes combined. In general, Lake Superior′s native ecosystem is relatively intact compared to the lower Great Lakes due to its upstream location [1]. Studies of the lake’s food web using stable isotope analysis [2], and distributions of fish [3], benthic invertebrates [4], and zooplankton [5] suggest it has nearshore and offshore components roughly divided by the 100 m depth contour. Recently, a trend towards reduced ice cover [6] has led to warmer summer surface temperature [7, 8] and higher wind speed [9]. As a result, the ranges of warm water species are expected to expand [10]. The goal of this study is to add to our understanding of how these physical changes may affect the lake by focusing on the variability of mixing and transport in between nearshore and offshore areas.

Circulation in Lake Superior varies on seasonal scales as a result of its dimictic cycle of vertical mixing and density stratification. For example, cross- shelf transport is inhibited during the onset of seasonal stratification in spring and fall by the thermal bar, a shore-parallel temperature front at 4°C, freshwater′s temperature of maximum density (T_md_) [11,12,13]. And later in summer, after stratification is established lakewide, nearshore waters are isolated from offshore by counterclockwise coastal circulation [14,15] that tends to concentrate nutrients and pollutants from the surrounding landscape in coastal waters [16]. We hypothesize that horizontal mixing processes such as coastal upwelling and downwelling [17], eddies [18,19] and instabilities associated with thermal fronts [20] reduce the trapping action of the dominant coastal flow and drive exchange between the lake’s nearshore and offshore areas. In addition, these processes are likely to affect nearshore ecosystems by controlling the distribution of plankton including fish larvae [21], phytoplankton [22] and zooplankton [23,24]. Despite their importance for nearshore ecosystems, the variability and scales over which these processes operate are not well understood.

In a previous study [25] we identified eddies in the nearshore areas of Lake Superior using a combination of satellite-based Synthetic Aperture Radar (SAR) and thermal imagery. Detection of eddies in SAR images is limited to low wind periods and also depends on the presence of surfactants, concentrations of which are expected to be generally low in the oligotrophic and relatively unpolluted surface waters of Lake Superior. Eddies were more frequently identified in images acquired in late summer, in areas where the thermal imagery showed a nearshore to offshore thermal gradient. The collection of SAR images did not include repeat passes that could have provided insight into eddy lifetimes, and no information on the vertical structure of the eddies was available. Thus, an additional goal of the work presented here was to improve our understanding of eddies in the lake. The following section briefly describes the two sets of model runs carried out in this study including a description of the tracers used. This is followed by results and discussion of each set of model runs and conclusions.

## Methods

### Model description and data sources

Lake Superior′s thermal structure and circulation was simulated using the Regional Ocean Modeling System (ROMS) [26], available at https://www.myroms.org/. ROMS is a free surface, hydrostatic primitive equations ocean model utilizing a terrain-following vertical coordinate system. It is widely used in applications covering a wide range of scales, including Lake Superior [27,28,29]. For this study, the model was configured for Lake Superior as described by White et al. [27]. It includes a fresh water equation of state, Mellor-Yamada 2.5 vertical turbulence closure scheme and the ROMS dynamic and thermodynamic ice module implemented in ROMS by Hedstrom [30]. The model of Matsumoto et al. [29], also derived from White et al. [27], contains modifications to the radiation code to produce more realistic ice. Those modifications were not used. The biological module used in the previous Lake Superior studies was also omitted.

A 2 km x 2 km horizontal grid was prepared using the grid utilities provided with the ROMS package and Lake Superior bathymetric data available at https://www.glerl.noaa.gov/data/char/bathymetry.html. The grid contained 16 terrain following vertical layers spaced unevenly throughout the water column so that vertical resolution was higher near the surface. The thickness of the surface sigma layer averaged 1.4 m whereas the thickness of the bottom layer averaged 22 m. The model was forced with meteorological data from the North American Regional Reanalysis (NARR) [31], available at http://www.esrl.noaa.gov/psd/data/gridded/data.narr.html.

Fluxes of latent and sensible heat as well as momentum were calculated within the model using the bulk flux option in ROMS. Radiative fluxes were input directly from the NARR, and shortwave radiation was attenuated in the water column using the Jerlov water type 1 parameter, corresponding to oligotrophic conditions. The ROMS uses a split-explicit time stepping scheme for computational efficiency. The baroclinic equations timestep was set to five minutes and the barotropic equations were solved 60 times between each baroclinic timestep. Daily averages of the model state were calculated internally and saved for analysis.

**Fig 1:**
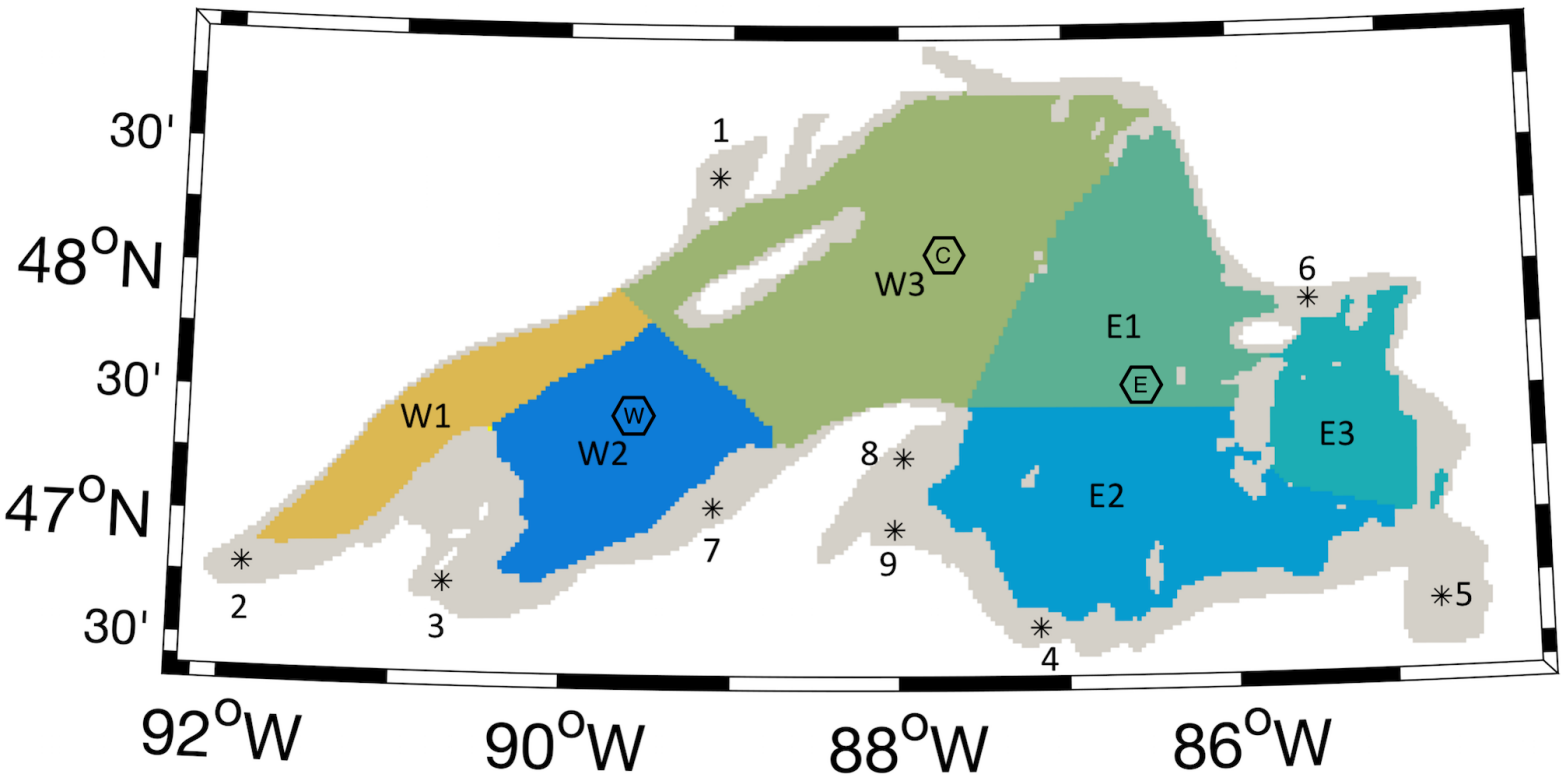
Nearshore and offshore areas of the model domain. The nearshore areas (gray) are where water depth is less than 100 m. Numbered points within the nearshore areas indicate the starting locations for passive dye tracers 1–9 used in the second set of model runs. The offshore areas are divided into western and eastern sectors identified by letter-number combinations discussed in the second set of model runs. The locations of the western (W), central (C) and eastern (E) NDBC meteorological buoys used for model validation are indicated by labeled polygons.

The National Oceanographic and Atmospheric Administration (NOAA) deploys meteorological buoys that measure meteorological data and surface water temperature at three locations in Lake Superior during the ice free months (Fig 1). Surface water temperatures at each buoy location covering 2003–2012 were downloaded from the National Data Buoy Center (NDBC) at http://www.ndbc.noaa.gov/ and visually compared to the 10 years of modeled surface temperatures for model validation.

### Simulations and tracers

We assessed mixing in Lake Superior’s nearshore areas using two sets of model runs. The first set simulated lake conditions over the 10 year period 2003–2012, and the second set focused on the summer of 2004. Each set of runs included its own group of virtual tracers. In the first, an aging tracer (hereafter *age)*, was used to measure residency time of water in the nearshore areas of Lake Superior (Fig 1). The concentration of *age*, expressed in units of time, increased with each timestep in each grid cell of the nearshore areas, while in the offshore areas, the concentration of *age* was set to a constant value of zero. *Age* was modified from Matsumoto et al. [30], who investigated Lake Superior’s vertical mixing timescale.

Here we use *age* to investigate rates of horizontal mixing between nearshore and offshore areas of the lake. *Age* is mixed between adjacent model cells at each timestep according to the model hydrodynamics, and does not mix between adjacent grid cells independently of the modeled circulation. For example, if no mixing occurred between the nearshore and offshore areas, the concentration of *age* would increase in each grid cell of the nearshore areas directly with, and always be equal to, the elapsed time of the model run. Mixing between the nearshore and offshore areas reduced *age* below this theoretical value. Although *age* could not exceed the total elapsed time of the simulation, mixing between a cell with low *age* and a neighboring cell with high *age* would increase *age* in the cell where *age* was initially lower by more than the intervening timestep.

The first set of model runs also included a passive ‘*nearshore tracer*’ (hereafter *nearshore*). The concentration of *nearshore* was set to a constant value of 100% in the nearshore areas and an initial concentration of 0% in the offshore areas. As the simulation progressed, the concentration of *nearshore* increased in the offshore areas as a result of mixing. The concentration of *nearshore* was reset to zero at the beginning of each year in the offshore areas.

The simulation started with current speed set to zero and a uniform temperature of 4°C throughout the model domain. NARR forcing for 2000, 2001 and 2002 was applied to initially set the seasonal pattern, followed by the NARR forcing for 2003–2010. The average daily *age*, *nearshore* and lake state variables including temperature and current speed were determined for the entire 10 year simulation and a linear regression was conducted to find significant (p value < .05) correlations between the interannual variability of *age* and the lake state variables.

In the second set of model runs, we focus on 2004. The model is run with nine passive tracers instead of *age* and *nearshore* for two 90-day periods, which start on April 1 and July 1 respectively. The objective of the 90 day runs is to compare alongshore and cross-shore dispersal in early summer, when only nearshore areas are stratified, to dispersal in late summer when the entire lake is stratified. Output from the 10 year simulation was visually inspected and 2004 was selected for the comparison because the transition to lakewide stratification in that year best matched the idealized case in which stratification develops in progressively deeper areas of the lake. Initial conditions were taken from the model state on May 31 and June 30, 2004 in the 10 year simulation. The concentration of the tracers was set to 100% throughout the water column at nearshore locations that each covered 10 grid cells, corresponding to an area of 200 km^2^ (Fig 1). Alongshore dispersal was determined as the change in tracer concentration in nearshore regions, and cross-shore dispersal was defined as change in by tracer concentration in offshore regions (Fig 1).

Heat content was estimated as

$$H=\sum_{i}\rho c_{p}T_{i}z_{i}A_{i}$$ (1)

where *ρ* is water density, taken to be 1000 kg m^-3^, *c*_*ρ*_ is the specific heat of water, taken to be 4180 J kg^-1^ K^-1^, and *T*_*i*_ is the temperature of a specific layer of thickness *z*_*i*_ and area *A*_*i*_. At each grid cell, heat content was calculated using the 16 layers of the model grid and grid cell area 4 km^2^. Surface heat flux was calculated internally within the model and output for analysis.

### Eddy detection algorithm

Eddies were identified in both sets of model runs using the Vector-Geometry Eddy Detection Algorithm v2.1, developed for use with ROMS by Nencioli et al. [32], who provide it freely to interested parties. The algorithm detects eddies based on the characteristic geometry of eddy circulation including a closed loop that has a velocity minimum near the eddy center and tangential velocity that increases approximately linearly with distance from the eddy center to a maximum value, beyond which it decreases.

The algorithm first checks the direction of the velocity vector at each grid cell of the surface velocity matrix. Locations where two neighboring cells have oppositely directed velocity vectors are identified as potential eddy centers. The velocity vectors at a user specified number of grid cells (parameter ‘*a*’) away from the potential eddy center are then checked to see if their directions are consistent with rotation around the potential eddy center. If this condition is satisfied, the grid cell with the lowest velocity within an area outlined by the closed circulation (parameter ‘*b*’) is identified as an eddy center. After eddy centers are located, the size and shape of the eddy is defined as the radius where the horizontal velocity shear changes sign. Several grid cells are required to resolve baroclinic eddy circulation in numerical models [33], and so parameter ‘*a*’, was set to three grid cells, and parameter ‘*b*’ was set to 2 grid cells. Although the circulation around eddy centers was a closed loop, the shape of the eddies was not necessarily symmetric about the center. Therefore, to describe the size of the eddies detected by the algorithm, the average of their east-west and north-south diameters was calculated.

We compared eddy sizes to the first baroclinic Rossby radius of deformation, using

$$R_{1}=\frac{\sqrt{{g\prime h}^{\prime }}}{f}$$ (2)

where *g*′ is reduced gravity, g(*ρ*_2_-*ρ*_1_)/*ρ*_2_, in which g is gravitational acceleration, 9.81 m s^-2^, and *ρ*_1_ and *ρ*_2_ are the densities of the surface and bottom layers; *h*′ is the effective mixed layer depth, h_1_h_2_/(h_1_ +h_2_), in which h_1_ and h_2_ are the thicknesses of the surface and bottom layer, and *f* is the Coriolis parameter at mid-latitudes, 10^-4^s^-1^.

At each eddy center, the depth of the maximum vertical temperature gradient in °C m^-1^ was determined by converting sigma levels to depths in meters, and calculating the vertical temperature gradient between discrete levels in °C m^-1^. The layer where the gradient was highest was used as the depth of the upper level, h_1_. The surface temperature at the same location was used to calculate *ρ*_1_ using the freshwater equation of state of Chen and Millero [34]. The difference between the depth of h_1_ and the center of the bottom sigma layer was used for h_2_, and the maximum density of freshwater [34] was used for *ρ*_2_. A threshold of 0.2°C m^-1^ was used to distinguish between stratified and unstratified conditions.

## Results and discussion

### Model—Data comparison

**Fig 2:**
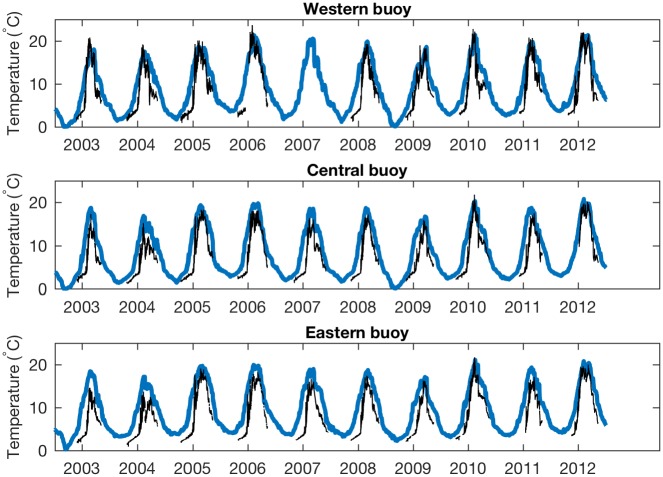
Modeled temperatures and NDBC buoy observations, 2003–2012. The continuous, year-round plot (blue) is model output. Buoy data (black) is only recorded during the summer period when the buoys are deployed. Data from the western buoy (top) is incomplete due to gaps in the observations.

Fig 2 compares model results to water temperatures measured at the three buoy locations over the 10-year simulation period. The model resolves Lake Superior’s dimictic thermal cycle in which surface temperatures pass through freshwater′s temperature of maximum density (T_md_), (approximately 4 °C), twice per year. The NARR forcing has the advantage of being easily accessed and widely used, however, it has a recognized warm bias over Lake Superior [16]. As a result of the model’s warm bias, ice formation in the model is lower than observed. Despite the model’s warm bias, it adequately resolves the variability of the lake’s seasonal cycle of mixing and stratification that is our primary focus.

### 10-year simulation, *age* and *nearshore* tracers

**Fig 3:**
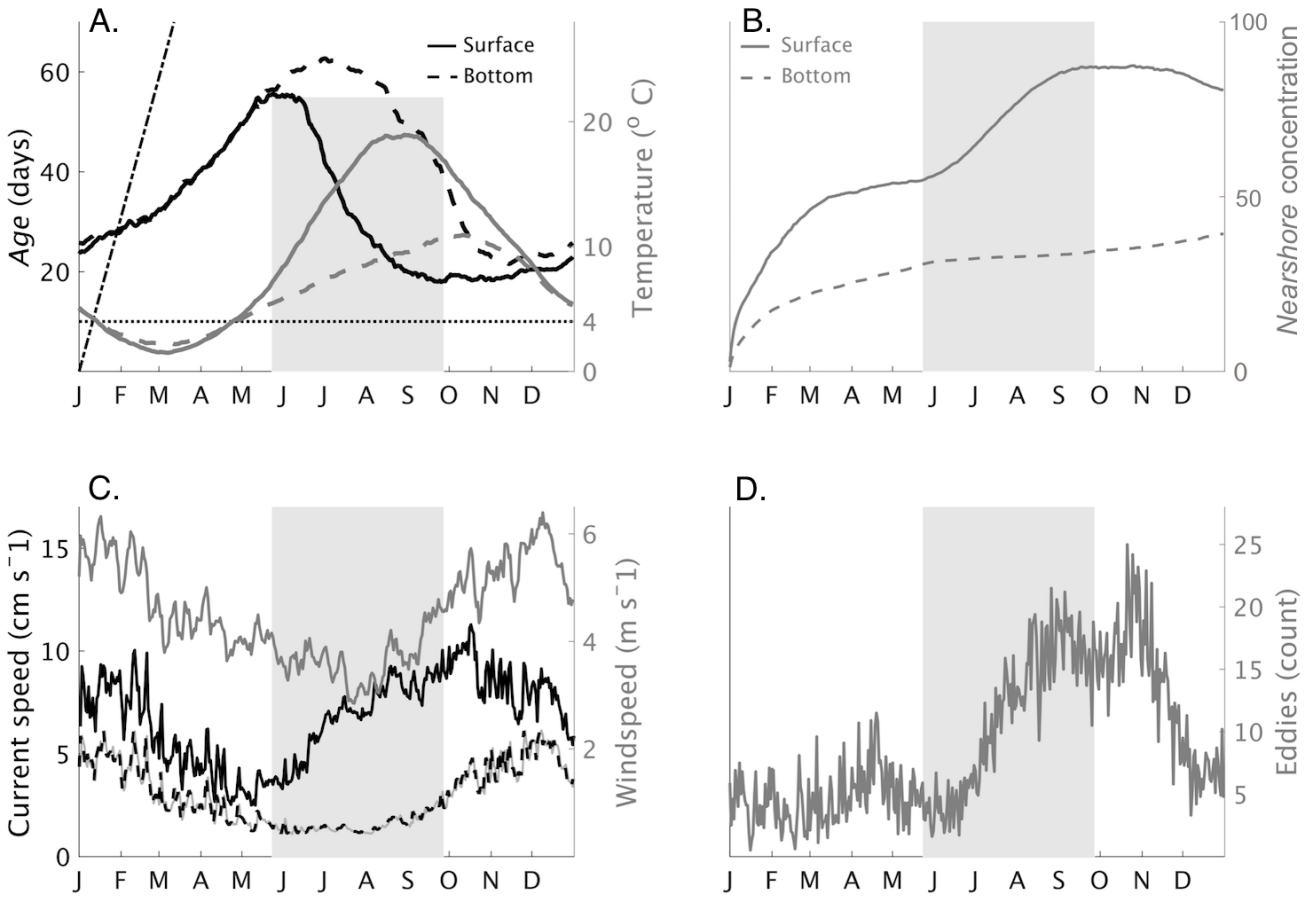
Average tracer and lake state variables, 2002–2013 simulation. (A) Daily average *age* and temperature in the nearshore areas. Surface (solid line) and bottom (dashed line) layer *age* are plotted in black (left axis). Surface and bottom layer temperature are plotted in gray (right axis). The average was calculated over the entire 10 years of model output and for the entire nearshore area of the model domain. The horizontal line at 4°C indicates freshwater′s temperature of maximum density. The dash-dotted line has a slope of 1 and indicates the rate at which *age* would increase with no mixing between nearshore and offshore areas. Shading indicates the time when *age* is decreasing. (B) Average *nearshore* concentration in the offshore area. The average was calculated over the entire offshore area. Shading indicates the time when *age* is decreasing. (C) Average current speed (left axis) in the surface layer (solid black line) and bottom layer (gray with black dashes). The average was calculated over the entire nearshore area and smoothed using a 15 day moving average filter. Shading indicates the time when *age* is decreasing. Also shown is wind speed (right axis, solid gray line). (D) The number of eddies detected by the algorithm over the entire model grid is shown. Shading indicates the time when *age* is decreasing.

Daily averages of *age* and *nearshore* tracer concentrations as well as lake state variables are shown in Fig 3. *Age* increased from late fall through spring and declined during the summer (black lines, Fig 3A, left axis). The rate of increase was below the slope of the hypothetical ‘no-exchange’ one-to-one aging line (straight dash-dotted line) indicating exchange between nearshore and offshore areas occurred year-round. *Age* values were similar in surface (solid line) and bottom (dashed line) layers until June, when surface layer *age* reached its annual maximum while bottom layer *age* continued to increase. Both surface and bottom layer *age* values declined during summer, and reached annual minimums in late September (surface) and November (bottom). The seasonal increase and decrease of *age* mirrored the dimictic cycle of vertical mixing and density stratification evident in the modeled temperatures (gray lines, Fig 3A, right axis). This included two periods when surface (solid line) and bottom (dashed line) layers were isothermal at T_md_ (horizontal line) as well as periods of density stratification in winter (February–March) and summer (July–October). Model surface temperatures in the nearshore area rose above T_md_ in early May.

The surface concentration of *nearshore* in the offshore area (Fig 3B) increased between January and March, and again between June and September, indicating nearshore-offshore exchange increased during these months. The difference in the dispersal of *nearshore* in the surface relative to its dispersal in the bottom layer is due to the effect of stratification. After stratification occurs, the surface and bottom layers decouple, and wind energy becomes focused in the surface layer, leading to relatively greater dispersal than in the bottom layer below the thermocline, where currents are slower. The increases in *nearshore* were consistent with the changes in *age* (Fig 3A). Between October and December, the concentration of *nearshore* decreased in the surface layer while simultaneously increasing in the bottom layer, an indication of vertical mixing. At the end of the year, the concentration of *nearshore* was less than one in the surface of the offshore area, and less than 0.5 in the bottom layer, indicating that mixing between nearshore and offshore areas was incomplete in a single year. This is consistent with the modeling work of Lam [35], who found the timescale of nearshore–offshore tracer exchange in Lake Superior was approximately three years.

Surface and bottom current speeds in the nearshore area were similar between November and May and fluctuated with changes in wind speed (Fig 3C). Between June and August, surface current speed increased sharply although wind speed remained low, reflecting the increase in density driven circulation during summer [16]. The number of eddies detected by the algorithm varied seasonally, with the minimum number occurring in spring, and the annual maximum occurring in November (Fig 3D).

**Fig 4:**
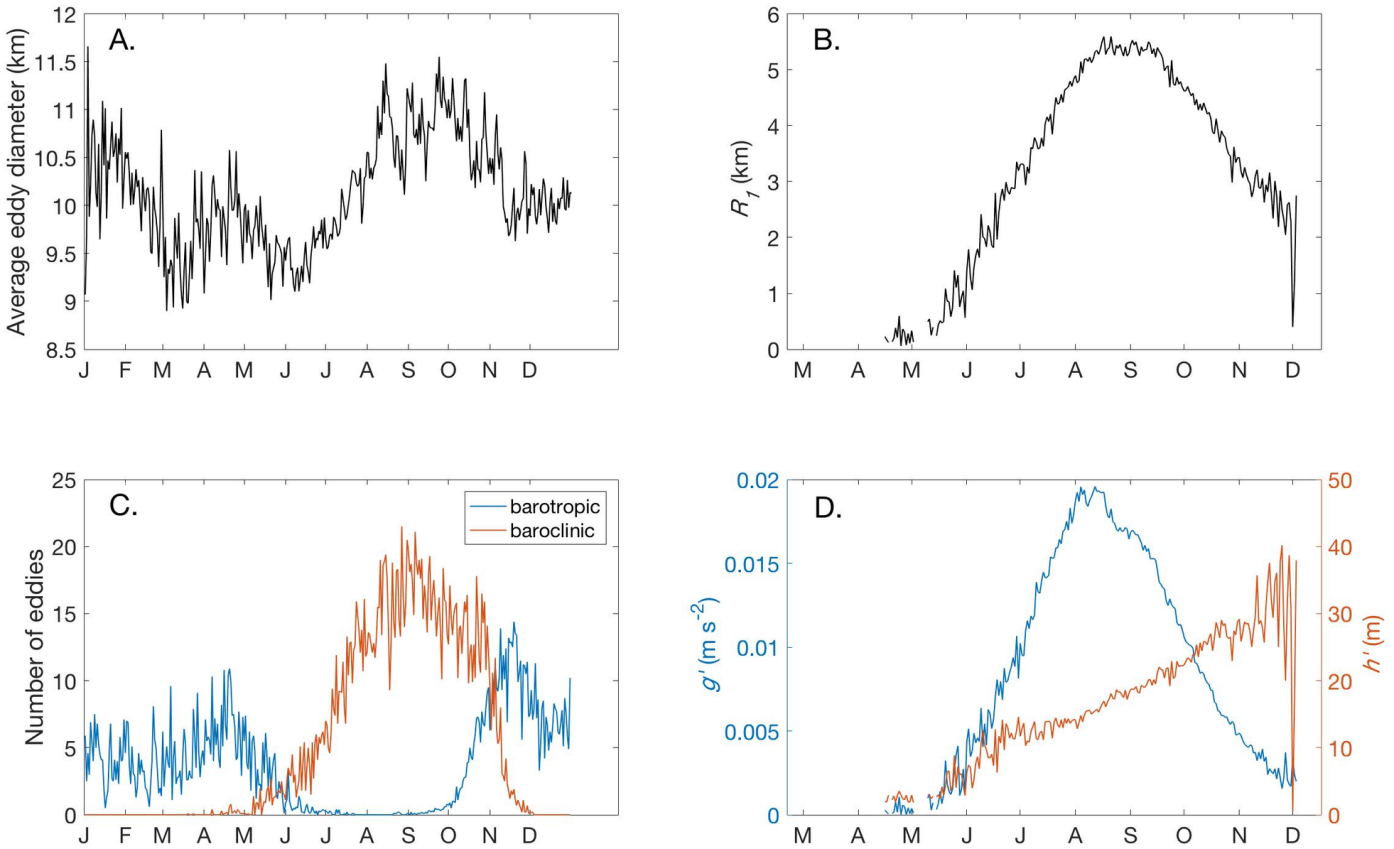
Eddy representation. (A) Eddy diameter was defined by the algorithm as the outermost closed streamline around an eddy center where velocity was still increasing. The plot shows the daily average of eddy diameter over the 10 year simulation, smoothed using a 15 day moving average. (B) Average baroclinic deformation radius *R*_*1*_ at eddy centers. *R*_*1*_ was calculated from model temperatures using a two-layer assumption of stratification. The daily average over the 10 year simulation is shown. (C) Daily average of barotropic and baroclinic eddies. Eddies were classified as barotropic (blue line) where the vertical temperature gradient was less than 0.2°C m^-1^. The daily average over the 10-year simulation is shown. (D) Average reduced gravity, *g*′, (left axis), and effective depth of the surface mixed layer, *h*′, (right axis) at eddy centers. The daily average of the 10-year simulation is shown.

In addition to increasing in number over the summer months, eddies increased in average size, from 9.1 km diameter in June to 11.4 km diameter in October (Fig 4A). *R*_*1*_ values calculated at eddy centers were defined only between May and November, when the water column was thermally stratified (Fig 4B). Given the symmetry of eddies, *R*_*1*_ values are expected to be half the eddy diameter. This was the case from July through September, when average *R*_*1*_ increased to a maximum of 5.6 km. The period from October through June included barotropic eddies for which *R*_*1*_ was not defined because the vertical temperature gradient beneath the eddy center was less than the 0.2°C m^-1^ threshold (blue line, Fig 4C).

*R*_*1*_ is a function of the vertical density gradient as well as the effective depth of the mixed layer (2). Both of these terms change during the course of the year in Lake Superior as part of the annual cycle of stratification and mixing. Fig 4D shows the values of *g*′ (left axis) and *h*′ (right axis) used in the calculation of *R*_*1*_ (Fig 4B). The early summer increase in *R*_*1*_ closely follows the increase in the stratification term, *g*′. The deepening of *h*′ in the fall offsets the decrease in *g*′ so that *R*_*1*_ remains near its annual maximum. The range of values for *h*′ is consistent with the observations of Austin and Allen [36], who found Lake Superior’s thermocline depth increased from 10 to 25 meters between July and October.

The diameter of eddies detected by the algorithm is comparable to the larger SAR-detected eddies in Lake Superior, which averaged 9.8 km in diameter [26]. Approximately 20% of the eddies identified in the SAR study were located within 7 km of shore. Because the algorithm checks circulation three grid cells away to define eddy centers, and the model grid has a horizontal resolution of 2 km, eddies resolved in the model whose centers lie within 7 km of shore are not detected by the algorithm. These could play a role nearshore–offshore exchange in areas where the 100 m bathymetric contour is within 7 km of shore, as along the western shore.

**Fig 5:**
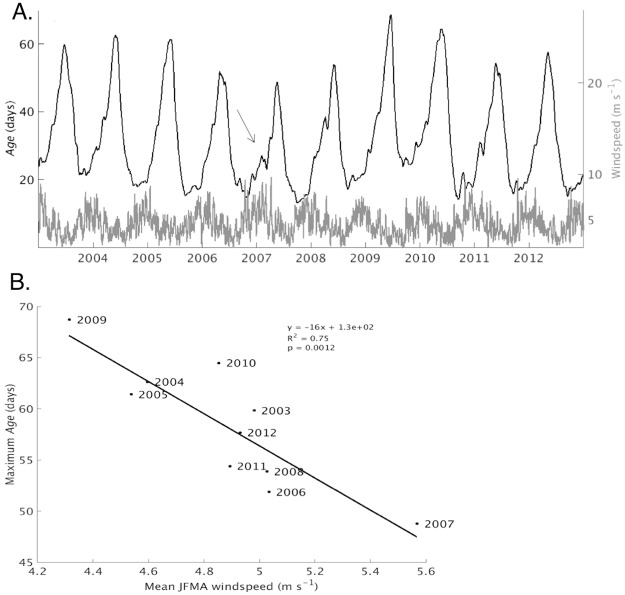
*Age* and wind speed comparison. (A) Daily *age* (black line, left axis) and wind speed (gray line, right axis) averaged over the entire nearshore region. The arrow indicates the sharp drop in *age* discussed in the text. (B) Correlation between the annual peak value of *age* shown in Fig 5A and average wind speed during late winter/early spring (J,F,M,A).

The following discussion pertains to values of *age* averaged across the entire nearshore region. The spatial variability of *age* is discussed separately below. Over the 10 year model run, values of *age* (Fig 5A, left axis) varied according to differences in the amount of mixing between nearshore and offshore areas. A comparison with lakewide average wind speed (Fig 5A, right axis) reveals that in most years, *age* is near its annual minimum at the beginning of the year, when wind speed is near its annual maximum. Over the first part of the year wind speed generally declines while *age* increases, although in some years the increase is interrupted by sharp drops corresponding to periods of high wind speed, for example, 2007 (Fig 5A, arrow). In contrast, the second half of the year is characterized by increasing wind speed and decreasing *age*.

As a metric for the total amount of mixing that has occurred during the first part of the year, we use the annual maximum of *age*. Fig 5B shows the correlation between *age* maximum value each year and average wind speed during the first four months of the year. The correlation is significant, with R-squared value of .75 and p-value of 0.001, suggesting average wind speed is a good predictor for the amount of mixing between nearshore and offshore areas in the first part of the year. Furthermore, relatively small differences in average wind speed correlated to dramatic differences in mixing, for example, the maximum was 40% lower in 2007, when the average wind speed was 5.6 m s^-1^, than in 2009, when the average wind speed was 4.3 m s^-1^. Overall, a one m s^-1^ increase in average wind speed correlated with a 16 day decrease in the maximum value of *age*. This sensitivity could be a result of the narrow shelf width over much of the nearshore area in Lake Superior (Fig 1).

Interannual variability of nearshore-offshore exchange could impact nearshore ecosystems by affecting the nearshore residence time of plankton in spring. For example, cisco, *Coregonus artedi*, spawn in nearshore areas of Lake Superior in late fall and their eggs overwinter and hatch there in spring when temperatures are favorable for rapid growth [37]. Previous research has shown that cisco recruitment is highly variable and sensitive to abiotic factors operating over regional scales [38]. In a study conducted in the productive waters along Lake Superior’s southern coast, Myers et al. found a negative correlation between peak wind speed and cisco recruitment [37]. Recruitment was low in 2007, a year with high peak wind speed, relative to 2009, a year with lower peak wind speed. Our results (Fig 5B) show the lowest *age* occurred in 2007, which also had the highest average springtime wind speed of any year in our 10-year simulation, whereas the highest *age* occurred in 2009, which had the lowest average wind speed. This supports the hypothesis that the correlation between wind speed and cisco recruitment is caused by longer nearshore residence time in years with lower springtime wind speed, allowing cisco to take advantage of the productive nearshore waters to a greater extent, leading to cisco recruitment success.

**Fig 6:**
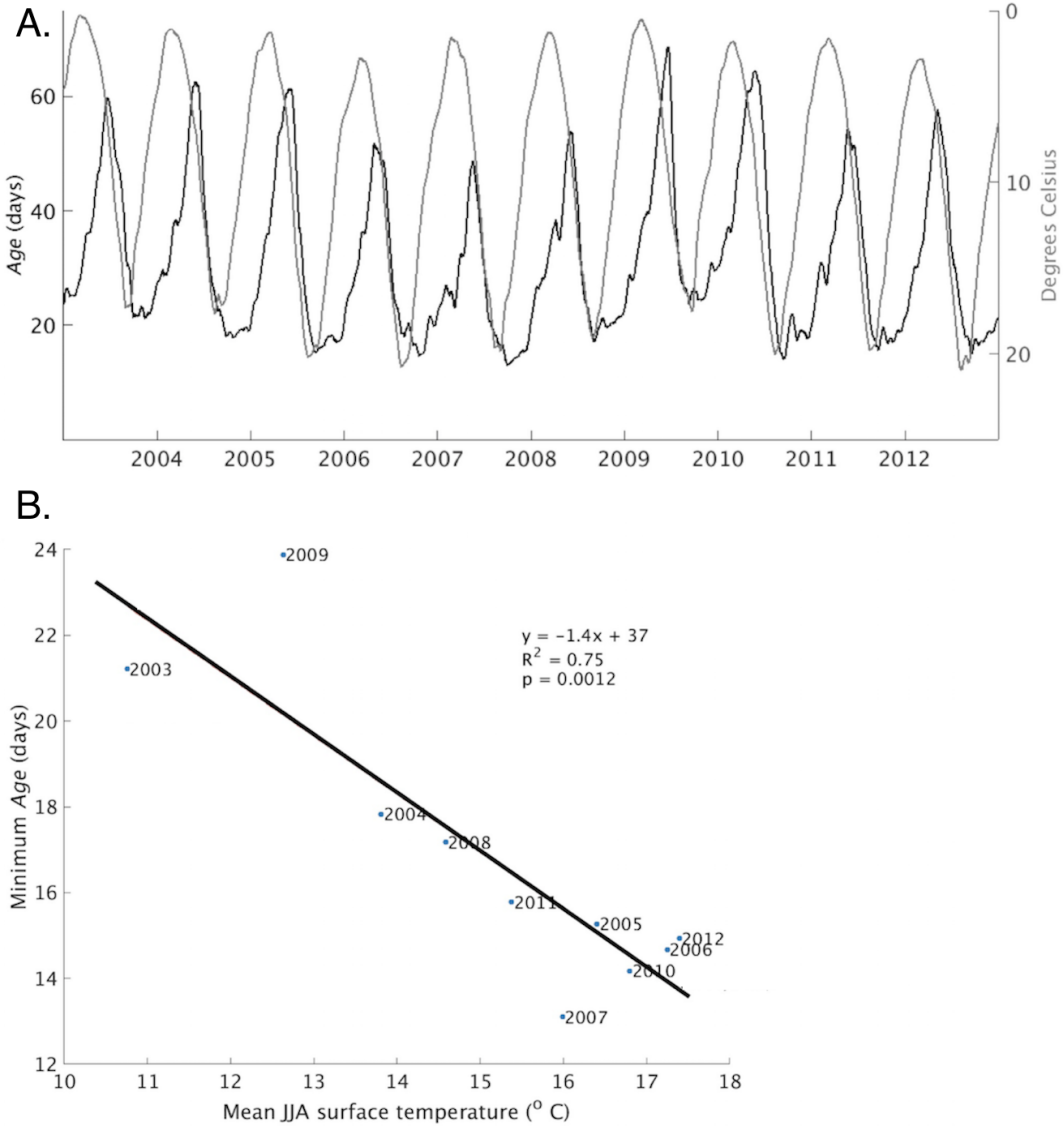
*Age* and surface temperature comparison. (A) *Age*, averaged over the entire nearshore area, is indicated by the black line and left axis. Surface temperature, averaged over the same area, is indicated by the gray line and right axis. The temperature axis is flipped vertically. (B) Correlation between the annual minimum of *age* and average summer (June, July and August) surface temperature. Shown is the equation and corresponding line resulting from a linear regression analysis.

Over annual scales, *age* (Fig 6A, black line, left axis) generally declined during periods when the average surface temperature in nearshore areas was increasing (Fig 6A, gray line, right axis). Fig 6B shows a significant negative correlation (R^2^ of 0.75 and p value of 0.0012) between the annual minimum *age* and the average summer (June, July, August) surface temperature, suggesting average summer surface temperature is a good predictor of the amount of mixing between nearshore and offshore areas. Furthermore, there was a dramatic difference between cold and warm years, with *age* values in cold years (2003, 2009) 50% greater than in warm years (2006, 2010). A one degree Celsius increase in the average temperature correlated to a 1.4 day decrease in the average *age*. The implication for nearshore foodwebs is that mixing between nearshore and offshore areas will be more extensive in years with warmer average summer surface temperatures relative to years with cooler summer surface temperatures, potentially reducing the nearshore residence time of plankton, a food source for nearshore benthos [4].

**Fig 7:**
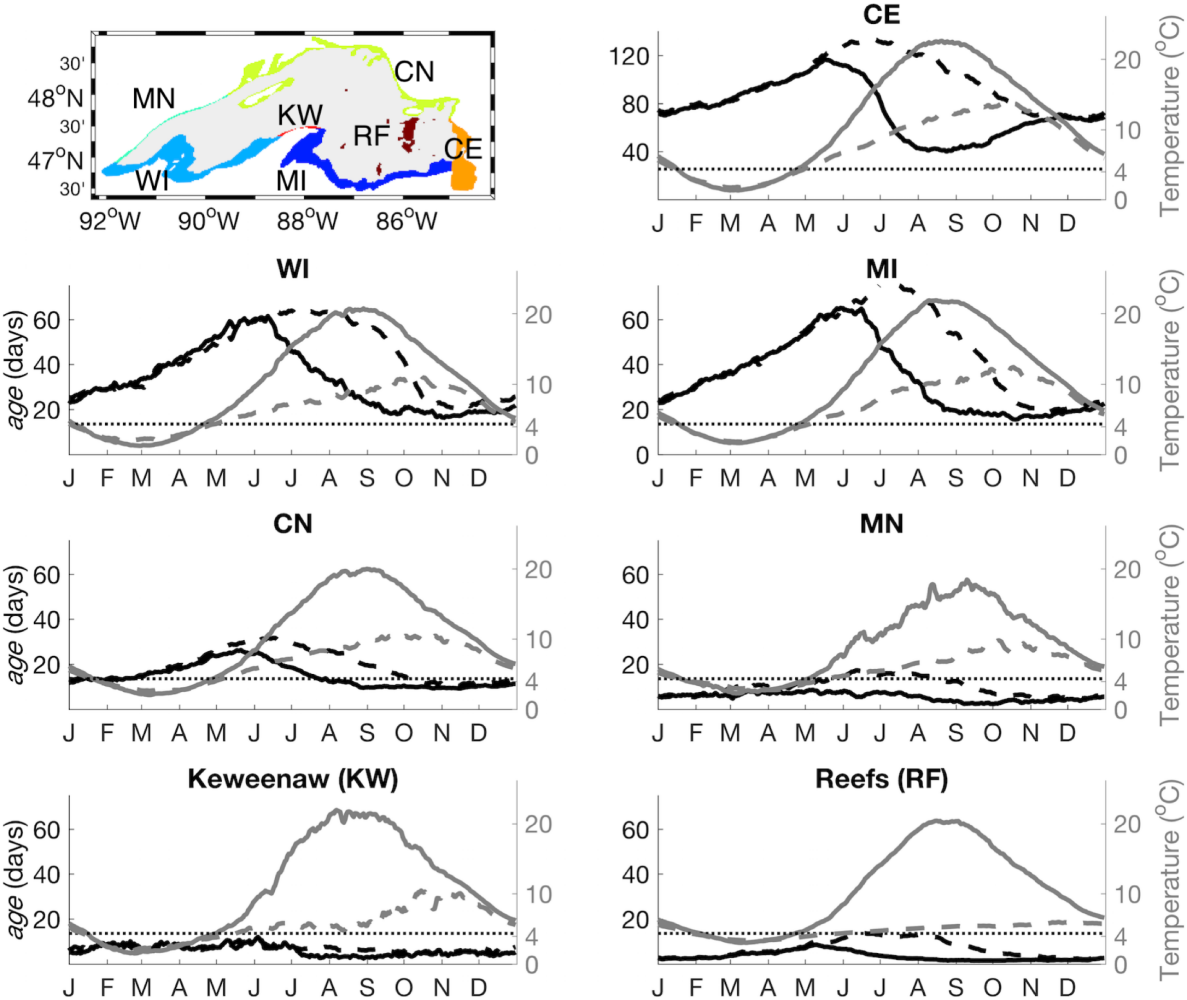
Average *age* and temperature for nearshore sectors. Daily average *age* (black lines, left axis) and temperature (gray lines, right axis). Surface values are plotted with solid line, bottom layer values are plotted with dashed line. Note the scale of the left-hand axis of the upper right panel is twice that of the other panels. The values were calculated over the area of each sector for the entire 2003–2012 simulation.

The values discussed above are lakewide averages. To determine the influence of shelf width on the values, the nearshore area was divided into sectors (Fig 7, Table 1). In the sectors where bottom slope is moderate (Table 1; MI, CE, WI, along the south and far east), *age* increased and decreased according to the seasonal cycle of stratification described above for the entire lake. In sectors where bottom slope is steep and shelf width narrow (Table 1; MN, KW), *age* was low year-round, indicating exchange between nearshore and offshore zones continued throughout the winter period. Expanding the width of the band classified as nearshore in these areas by increasing the water depth defining the nearshore or another method would provide more information on nearshore—offshore exchange in these sectors. The high value of *age* in the eastern Canada sector (CE) was due to the inclusion in that sector of the isolated large embayment in the southeast (Whitefish Bay), which had the highest value of *age* of any area in the lake.

**Table 1:** Shelf widths and bottom slopes for the nearshore sectors.

Nearshore Sector	Average shelf width	Average Bottom Slope
Wisconsin (WI)	17 km	0.006
Canada East (CE)	15 km	0.007
Michigan (MI)	14 km	0.007
Canada North (CN)	10 km	0.010
Keweenaw (KW)	3 km	0.033
Minnesota (MN)	2 km	0.050
Reefs (RF)		not defined (no shore)

### Summer stratified season simulation, regional tracers

**Fig 8:**
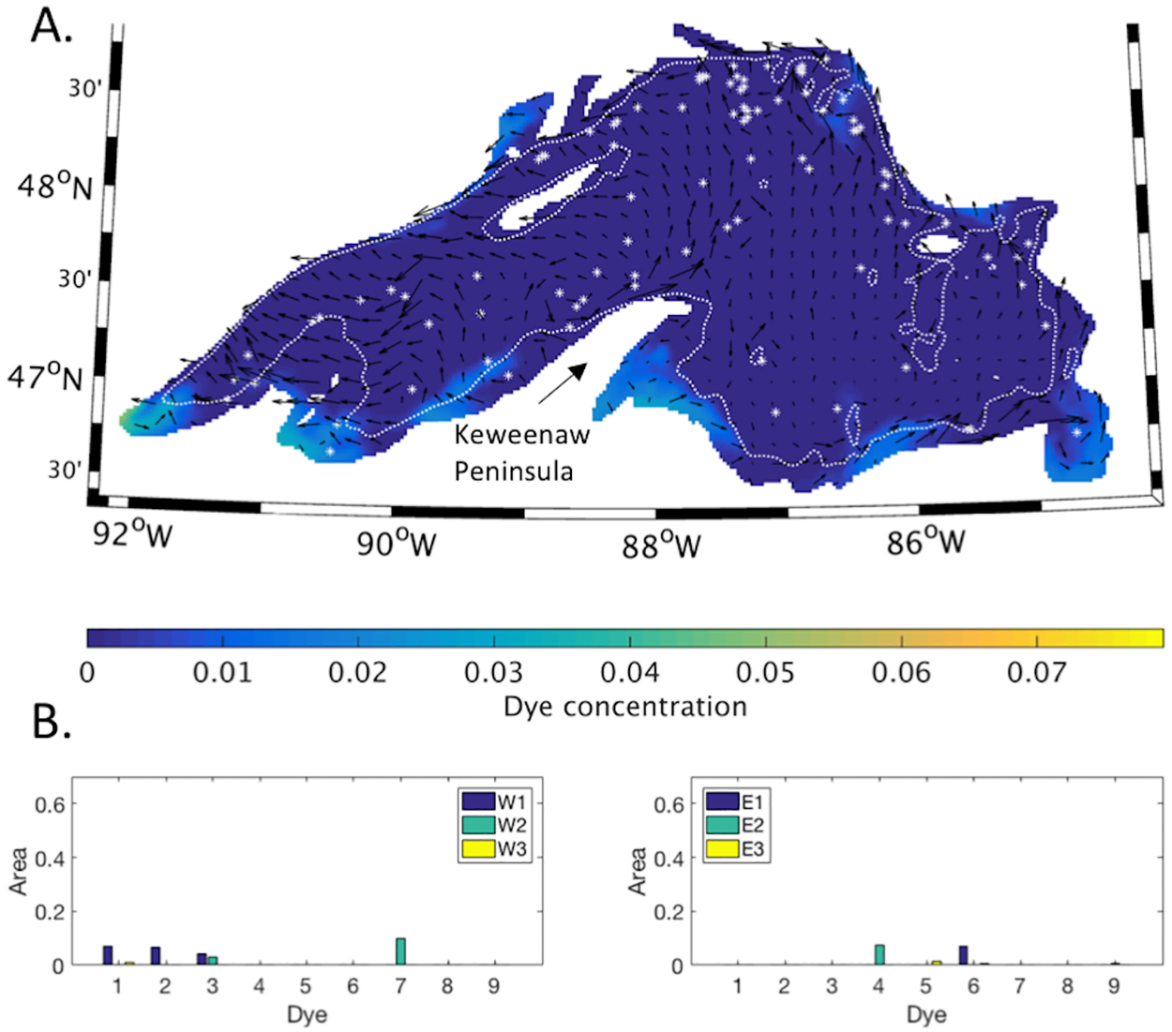
Early summer season dispersal of passive tracers. **(A)** Shading indicates surface concentration of passive tracers released on April 1, after 60 days. Vectors show the average direction of currents over the preceding 30 days. White stars indicate eddy locations over the previous 30 days. The white dotted line indicates the location of the 100 m depth contour. **(B)** The fraction of each offshore region where surface tracer concentration exceeds 0.01. Locations where tracers 1–9 were released are indicated in Fig 1.

The second set of model runs consisted of two simulations of 90 day periods in the summer of 2004. In the simulation which began on April 1, dispersal of the passive tracers was generally limited to small areas adjacent to their release points (Fig 8A). The tracers with the greatest offshore dispersal were tracers 1–3, which were released in the western nearshore regions (Fig 8B). Over the 90 day simulation, a total of 400 eddies were detected, of which 320 were located in the offshore regions. The offshore region with the highest number of eddies relative to the size of the region was w3 (S1 Table). There eddies were concentrated within the mean circulation connecting the Keweenaw current and the northern shore (Fig 8A). The nearshore region with the highest number of eddies relative to size was region WI.

**Fig 9:**
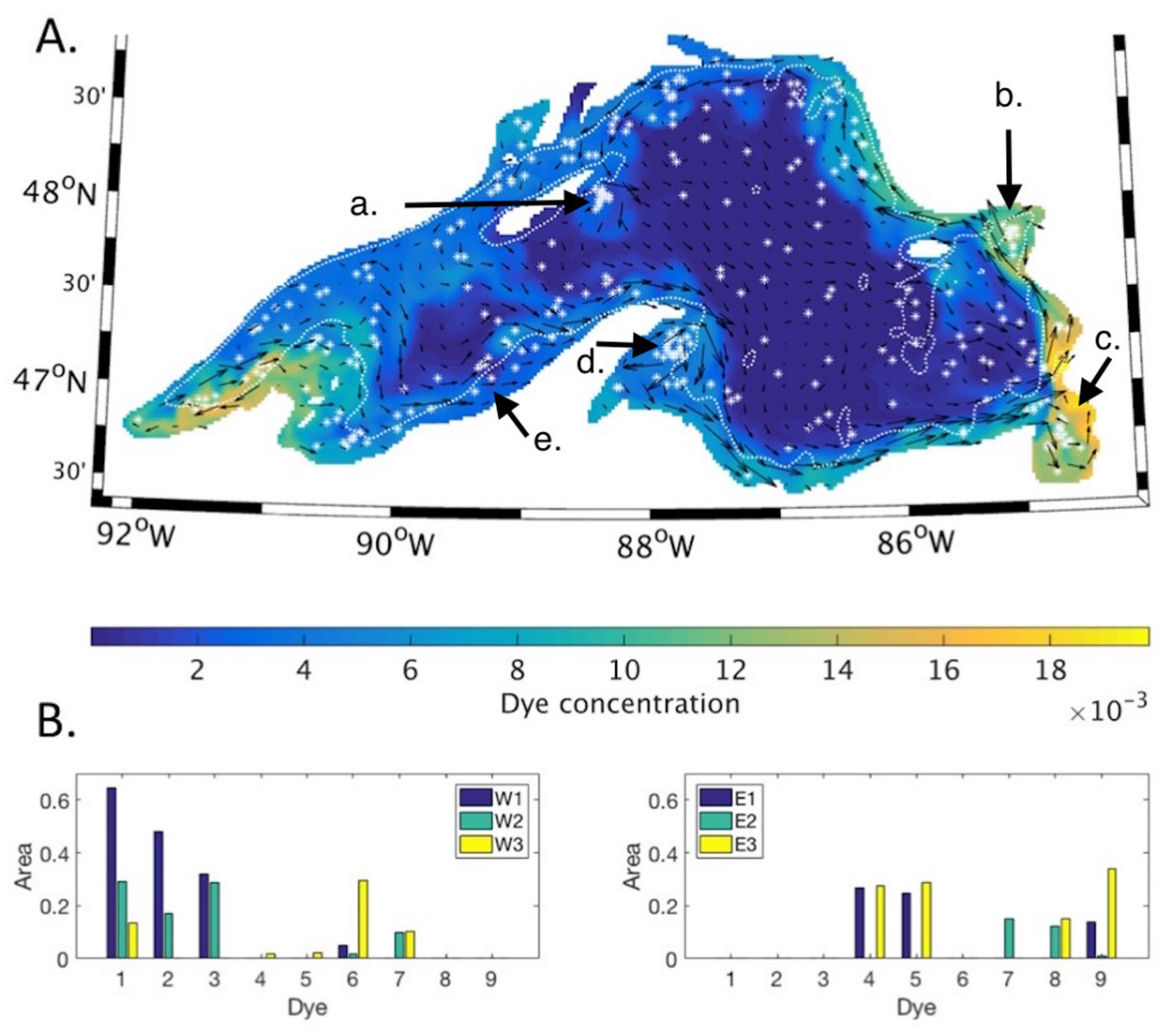
Late summer season dispersal of passive tracers. **(A)** Shading indicates surface concentration of passive tracers released on July 1, after 60 days. Vectors show the average direction of currents over the preceding 30 days. White stars indicate eddy locations over the previous 30 days. Arrows a-e indicate areas where eddies were detected in the SAR study discussed in the text. The white dotted line indicates the location of the 100 m depth contour. **(B)** The fraction of each offshore region where surface tracer concentration exceeds 0.01. Locations where tracers 1–9 were released are indicated in Fig 1.

In contrast, in the simulation that began on July 1, alongshore and cross shore dispersal of the tracers was extensive and reflected coastal circulation in the south and east, as well as cross-basin circulation in the west (Fig 9A). Seven of the nine passive tracers were dispersed into the western offshore regions (Fig 9B), including those initially released in the eastern nearshore regions (tracers 4–6, Fig 1). Tracers initially released in the western nearshore regions remained in the western basin and were not dispersed into the eastern offshore regions. The number of eddies increased to 1052 in total, of which 723 were located in the offshore regions (S1 Table). The offshore region with the highest number of eddies relative to its size was region w1, where eddies were concentrated along the 100 m bathymetric contour. In addition, eddies were detected in locations where they had been identified previously in SAR imagery [26] (Fig 9Aa–9Ae).

These patterns may provide pathways for nearshore and offshore distribution of plankton species. For example, Watson and Wilson [39] suggested the late summer basin scale circulation of Lake Superior [16], disperses zooplankton from embayments in the south and east into northwestern offshore areas. In offshore areas, and over smaller spatial scales, they found patchy zooplankton abundance, correlated with features identified in an associated study as internal waves [40]. These characterize the lake’s offshore circulation in summer [41]. Megard et al. [24], found zooplankton aggregations in the western offshore regions were correlated with eddies that formed during upwelling events.

**Fig 10:**
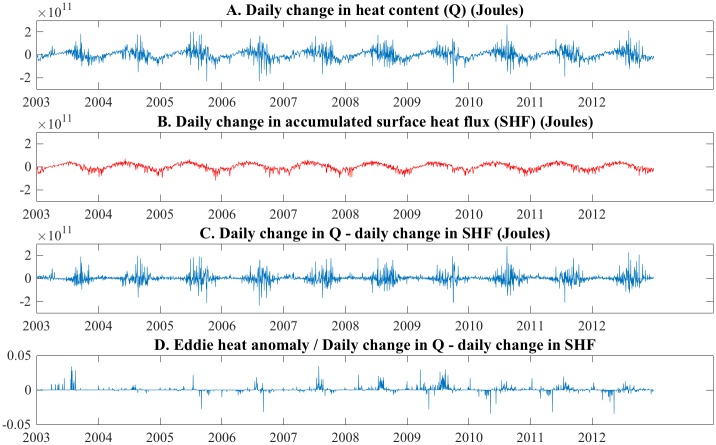
Daily change in heat content and surface heat flux, nearshore sector WI. Changes in daily heat content (A), surface heat flux (B), and their difference (C), and daily heat anomaly due to eddies (D).

To estimate the contribution of eddies to mixing between nearshore and offshore regions, we used the eddies detected by the algorithm as an objective measure of eddies resolved in the model, and compared their combined heat content to the total lateral heat transport between the WI nearshore region (Fig 7A), and its offshore neighbors. The passive tracer results indicated the WI nearshore region was an area of active mixing, and also had a relatively high number of eddies detected by the algorithm. The daily change in heat content of the region was calculated (Fig 10A), and the contribution from surface heat flux (Fig 10B) was subtracted to yield the change in heat content due to lateral transport. The results (Fig 10C) show changes in heat content during roughly the first half of each year were primarily due to surface heat flux, and the contribution from lateral transport was near zero. Lateral transports increased dramatically in the second half of each year, due to rapid fluctuations.

To evaluate whether eddies contributed to the fluctuations in the second half of the year (Fig 10C), the heat content of individual eddies detected in the region was compared to the fluctuations. Eddy heat content was determined by calculating their volume using the eddy diameter and a mixed layer depth of 10 m, which was used for all eddies. The heat anomaly of individual eddies was calculated (2) and summed to give the total daily heat anomaly due to eddies. Fig 10D shows the daily heat anomaly due to eddies is a few percent of the total lateral heat exchange shown in Fig 10C. Earlier (Fig 4D) we showed the mixed layer deepens from 10 to 25 m depth in late summer, which would increase the eddy contribution up to approximately 10 percent by increasing eddy volume and heat content. This is likely a conservative estimate given the limitations of the eddy detection algorithm.

## Summary and conclusions

The nearshore areas of large lakes are valuable water resources and habitat where water quality is affected by inputs from the surrounding watershed as well as horizontal mixing processes that disperse pollutants, nutrients and sediment along shore and across shore. In this study, horizontal mixing in Lake Superior was investigated using a realistically forced 3 dimensional numerical model and virtual tracers. Two sets of model runs were completed. The first was a simulation of lake conditions over the ten year period 2003–2012 which focused on mixing between nearshore areas where water depth is less than 100 m and deeper offshore areas. The concentration of an aging tracer was used to measure residency time of water in the nearshore areas. Mixing, indicated by changes in tracer concentration, was weaker during winter and spring when the lake was isothermal or weakly stratified than in summer after seasonal density stratification was established.

Interannual differences in the amount of mixing in the winter/spring period were negatively correlated with average wind speed between January and April. Over the time period simulated, the amount of mixing, expressed as tracer concentration, was greater years with higher average wind speed, reducing nearshore *age* by 40% compared to years with lower average windspeed. In some years, the spring mixing was dominated by brief periods of high wind speeds, supporting the hypothesis that high wind speed associated with spring storms reduces the residence time of plankton in nearshore areas. Interannual differences in summer were significantly correlated to the average summer surface temperature. The results indicate exchange between the nearshore and offshore areas of Lake Superior can be expected to increase under a trend of earlier stratification and warmer summer surface temperatures.

The second set of model runs compared the dispersal of passive tracers released from nine locations around the model lake’s perimeter in early summer to their dispersal in late summer. Dispersal was more extensive in the late summer simulation, when density stratification was established lakewide. In the eastern lake basin, dispersal was primarily alongshore due to counterclockwise coastal circulation. In the western basin, dispersal was primarily offshore due to upwelling and cross-shore currents. The different dispersal patterns were also evident in the distribution of eddies resolved in the model. Analysis of surface circulation showed eddies formed in the western basin during low wind periods following upwelling events whereas in the eastern basin, eddies formed in bays and embayments where the coastal current interacted with topographic features including headlands. This included several areas where they had been identified previously in satellite images.

## Supporting information

S1 Table (DOCX)

S1 Data (MAT)
