# Supplementary material for: Modeling nearshore-offshore exchange in Lake Superior
Source: PLoS One. 2018 Feb 15;13(2):e0193183. doi: 10.1371/journal.pone.0193183 (PMC5814091; doi:10.1371/journal.pone.0193183)
Supplement: S1 Table — (DOCX) [file pone.0193183.s001.docx]

| Nearshore | Eddies; Apr-Jun | Density | Eddies; Jul - Sep | Density |
| --- | --- | --- | --- | --- |
| WI | 35 | 0.021 | 108 | 0.065 |
| MI | 15 | 0.010 | 97 | 0.067 |
| ER | 5 | 0.017 | 11 | 0.037 |
| EC | 14 | 0.019 | 72 | 0.096 |
| NC | 11 | 0.008 | 41 | 0.030 |
| NM | 0 | 0.000 | 0 | 0.000 |
| KW | 0 | 0.000 | 0 | 0.000 |

| Offshore | Eddies; Apr-Jun | Density | Eddies; Jul - Sep | Density |
| --- | --- | --- | --- | --- |
| w1 | 29 | 0.022 | 139 | 0.107 |
| w2 | 36 | 0.018 | 96 | 0.049 |
| w3 | 123 | 0.027 | 235 | 0.051 |
| e1 | 46 | 0.015 | 60 | 0.019 |
| e2 | 64 | 0.021 | 103 | 0.033 |
| e3 | 22 | 0.019 | 90 | 0.076 |
